# The 'Walking for Wellbeing in the West' randomised controlled trial of a pedometer-based walking programme in combination with physical activity consultation with 12 month follow-up: rationale and study design

**DOI:** 10.1186/1471-2458-8-259

**Published:** 2008-07-26

**Authors:** Claire F Fitzsimons, Graham Baker, Annemarie Wright, Myra A Nimmo, Catharine Ward Thompson, Ruth Lowry, Catherine Millington, Rebecca Shaw, Elisabeth Fenwick, David Ogilvie, Joanna Inchley, Charlie E Foster, Nanette Mutrie

**Affiliations:** 1 Department of Sport, Culture and the Arts, University of Strathclyde, 76 Southbrae Drive, Glasgow, G13 1PP, UK; 2 Strathclyde Institute of Pharmacy and Biomedical Sciences, University of Strathclyde, The John Arbuthnott Building, 27 Taylor Street, Glasgow, G4 0NR, UK; 3 OPENspace, Edinburgh College of Art, Lauriston Place, Edinburgh, EH3 9DF, UK; 4 Public Health and Health Policy, Division of Community Based Sciences, University of Glasgow, 1 Lilybank Gardens, Glasgow, G12 8RZ, UK; 5 MRC Epidemiology Unit, Institute of Metabolic Science, Box 285, Addenbrooke's Hospital, Hills Road, Cambridge, CB2 0QQ, UK; 6 CAHRU, Moray House School of Education, University of Edinburgh, St Leonard's Land, Holyrood Road, Edinburgh, EH8 8AQ, UK; 7 Division of Public Health and Primary Health Care, Rosemary Rue Building, Old Campus Road, Roosevelt Drive, Headington, Oxford, OX3 7LF, UK; 8 School of Sport and Exercise Sciences, University of Loughborough, UK

## Abstract

**Background:**

Scotland has a policy aimed at increasing physical activity levels in the population, but evidence on how to achieve this is still developing. Studies that focus on encouraging real world participants to start physical activity in their settings are needed. The Walking for Well-being in the West study was designed to assess the effectiveness of a pedometer-based walking programme in combination with physical activity consultation. The study was multi-disciplinary and based in the community. Walking for Well-being in the West investigated whether Scottish men and women, who were not achieving the current physical activity recommendation, increased and maintained walking behaviour over a 12 month period. This paper outlines the rationale and design of this innovative and pragmatic study.

**Methods:**

Participants were randomised into two groups: Group 1: Intervention (pedometer-based walking programme combined with a series of physical activity consultations); Group 2: Waiting list control for 12 weeks (followed by minimal pedometer-based intervention). Physical activity (primary outcome) was measured using pedometer step counts (7 day) and the International Physical Activity Questionnaire (long version). Psychological processes were measured using questionnaires relating to the Transtheoretical Model of Behaviour Change, mood (Positive and Negative Affect Schedule) and quality of life (Euroqol EQ-5D instrument). Physiological measures included anthropometric and metabolic outcomes. Environmental influences were assessed subjectively (Neighbourhood Quality of Life Survey) and objectively (neighbourhood audit tool and GIS mapping). The qualitative evaluation employed observation, semi-structured interviews and focus groups. A supplementary study undertook an economic evaluation.

**Discussion:**

Data analysis is on-going. Walking for Well-being in the West will demonstrate if a pedometer based walking programme, in combination with physical activity consultation results in a sustainable increase in walking behaviour in this sample of Scottish adults over a 12 month period. The study will examine the complex relationships between behavioural change, health consequences and the role of the environment, in conjunction with the cost effectiveness of this approach and a detailed insight into the participants' experiences of the intervention.

**Trial registration:**

Current Controlled Trials ISRCTN88907382

## Background

Scotland's national physical activity strategy 'let's make Scotland more active' set out to improve national physical activity levels [1]. The strategy highlighted walking as an ideal mode of activity as it does not require any special planning, clothing or skills. Walking has been shown to be a popular mode of physical activity both within Scotland [2] and in the European Union as a whole [3]. Mutrie and Hannah [4] recently showed that, for a representative sample of the population of the West of Scotland, the percentage of people walking was similar in younger, middle-aged and older age groups, whereas participation in other physical activities showed a marked decline with age. In addition there was less difference, both between men and women and between affluent and less affluent groups, in the proportion of people walking than in the proportion of people doing other physical activities. Within Scotland the proportion of adults not meeting the current physical activity recommendation (30 minutes of at least moderate intensity activity on at least five days of the week [5]) is highest in the most deprived areas (defined as the 5^th ^quintile in the Scottish Index of Multiple Deprivation), where 35% of men and 26% of women achieve the recommendation, compared with 41% and 32% respectively in the least deprived areas (1^st ^quintile) [6]. Redressing health inequalities such as this has become a central component of Scottish policy [7].

A recent systematic review [8] synthesised the findings of controlled before and after studies of interventions to promote walking. From the findings of 19 randomised controlled trials and 29 non-randomised controlled studies, the reviewers concluded that motivated individuals can be encouraged to walk more by targeted, tailored interventions delivered at the level of the individual, household or group. However, Ogilvie *et al.*, concluded the sustainability, generalisability and health benefits of many of the approaches investigated in the review remain to be convincingly demonstrated. The review found much of the evidence to date on the use of pedometers has been collected from studies based in the USA with relatively small sample sizes and short follow-up periods, sometimes a few weeks. There was limited evidence on the ability of pedometers to sustain an increase in walking over the longer term. Bravata *et al.*, [9] recently carried out a systematic review which looked specifically at the use of pedometers to increase physical activity. They also concluded the long term effects of pedometers remain undetermined. Of the four pedometer studies included in the recent NICE guidelines on the promotion of physical activity, the longest follow-up was at 24 weeks [10]. Despite the appeal of walking as a mode of physical activity, large knowledge gaps exist on the optimum methods to promote and sustain walking behaviour.

Using the information from the Ogilvie *et al.*, systematic review [8], which was conducted by our research group, we designed a randomised controlled trial of an intervention to promote walking in 18–65 year old men and women in a community in the west of Glasgow (Walking for Well-being in the West (WWW), start date August 2006). WWW was designed to assess whether a pedometer-based walking programme, in combination with physical activity consultation, would increase and sustain independent walking over 12 months in adults who are not meeting the current physical activity recommendation. The WWW study was designed as a multi-disciplinary study to investigate the behavioural, psychological and physiological consequences of the intervention, in conjunction with an assessment of how an individual's local environment influences their walking. In addition, a qualitative evaluation explored participants' and researchers' experiences of the intervention. A supplementary study carried out an economic evaluation to assess cost-effectiveness of the intervention. This type of evaluation is vital to illuminate the real impact of the study on health behaviour and include the potential effects of place and social conditions [11,12].

In our paper we present the rationale and design of each evaluation component of the study and discuss the study's potential contribution to the evidence base for physical activity promotion.

## Methods

### Aim

This randomised controlled trial was pragmatically designed to assess the effectiveness of a pedometer-based walking programme, in combination with physical activity consultation at increasing and maintaining walking behaviour over a 12 month period. In addition the study would also evaluate the potential mechanism for physical activity behaviour, the impact of individual and environmental determinants, the health benefits, economic costs and participants' experiences of the study.

### Ethical Approval

Appropriate ethical approval was sought from the University of Strathclyde ethics committee and all procedures were carried out in accordance with the Declaration of Helsinki.

### Recruitment process

Recruitment to the WWW trial took place between August and December 2006. Recruitment was targeted specifically at low active individuals in the lowest socio-economic groups. To assess the extent of deprivation in the study area the Scottish Index of Multiple Deprivation (SIMD) was used. The SIMD is the official measure of relative area based deprivation in Scotland and is based on 37 deprivation indicators across 7 domains: current income, employment, housing, health, education, skills and training, and geographical access to services and telecommunications [13]. These measures are used to split the country into data zones of between 500 and 1000 people, which are then ranked from the most deprived (1) to least deprived (6505) on the overall SIMD index.

The first phase of recruitment involved 4 data zones within 1 km radius of the university campus that were classified in the top 15% of the SIMD statistics (i.e. the most deprived), along with an additional zone the centre of which was within 1 km of the campus. These zones were selected to maximise ease of access to the campus and minimise participant burden when attending appointments. The second phase of recruitment involved 9 data zones in the same deprivation category that fell within a 1.5 km radius of the campus (4 of these were partially within the 1 km boundary). The third phase of recruitment included an additional 10 data zones that fell within a 1.5 km radius of the campus (regardless of SIMD category but again selected to maximise ease of access to the campus). All households received a leaflet advertising the project. Posters and leaflets were also placed in GP surgeries, other health care providers, shops, veterinary practices and pubs. Community stands in the local library, shopping centre and high-rise blocks of flats further advertised the project. The project was also advertised through the local newspaper. The study area was urban with predominant land-use being residential.

### Study population

Men and women were eligible to enter the trial if they were aged 18–65 years, able to understand the rationale behind the trial, were able to walk independently for 5–10 minutes, spoke English, and were in the precontemplation, contemplation or preparation stages of the transtheoretical model of behaviour change (with respect to meeting the current physical activity recommendations) using an adapted stage of change algorithm [14]:

Stage 1: Precontemplation: I am not regularly physically active and do not intend to be so in the next 6 months

Stage 2: Contemplation: I am not regularly physically active but am thinking about starting to do so in the next 6 months

Stage 3: Preparation: I do some physical activity but do not take part in regular physical activity

Potential participants were excluded if they were involved in regular activity (i.e. not in stages 1–3 of the transtheoretical model of behavioural change). All participants were screened using the Physical Activity Readiness Questionnaire [15] to identify contraindications to physical activity. Any individual with a possible contraindication to an increased level of physical activity was referred to their general practitioner for approval before participation in the study was allowed.

Written informed consent was obtained in five sections: 1. Study participation, study questionnaires and pedometer use; 2. Body composition measures: height, weight, waist circumference, skinfold thickness; blood pressure, heart rate; 3. Provision of a blood sample; 4. Participation in focus group; 5. Video recording of a proportion of physical activity consultations. To be included in the study participants were required to consent to Section 1 but had the opportunity to opt out of Sections 2–5.

### Randomisation

The participants were stratified by baseline step count (average daily step count ≤ 8000 steps/day vs. > 8000 steps/day) and gender and then randomised into one of two groups: immediate intervention (group 1) or waiting list control (group 2). The value of 8,000 steps was used as a stratification variable to account for individuals with a high baseline step-count. This value has previously been used as a baseline descriptor for sedentarism [16]. Researchers have also suggested that individuals are more likely to attain public health guidelines by walking at least 8000 steps/day [17]. Positive effects on conventional metabolic parameters, such as blood pressure, have been found when steps are above 8000 steps/day [18]. We chose not to exclude individuals with daily step counts above a certain value as the activity may have consisted solely or primarily of incidental activity. In addition, we did not wish to exclude individuals who had classified themselves as inactive via the stage of change algorithm which corresponds to the public health guidelines.

**Figure 1:**
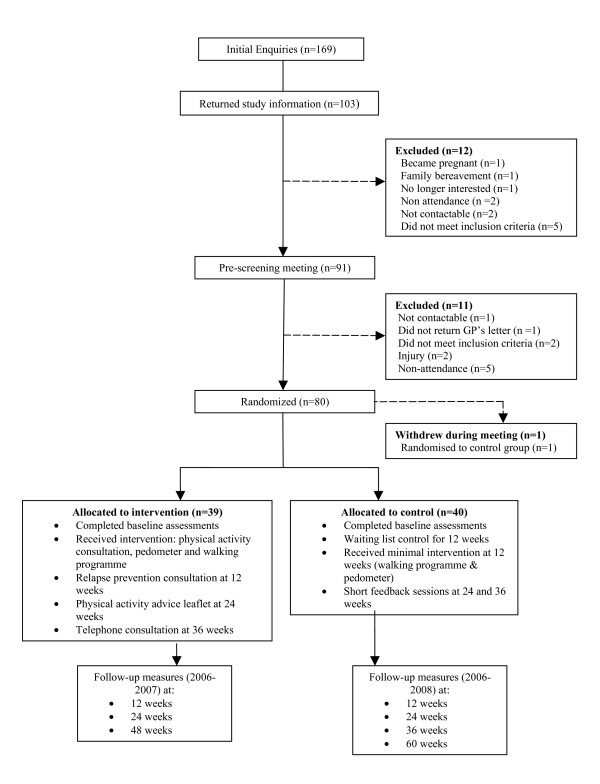
Flowchart of participant recruitment and trial design.

Baseline step counts were measured using a sealed Omron HJ-109-E pedometer (Omron Healthcare UK Ltd) over a 7 day period. Randomisation was carried out via an independent interactive voice response telephone system. Researchers who conducted the physical activity consultations could not be blinded to group allocation and they therefore informed the participants which group the telephone system had allocated them to. Researchers performing the physiological assessments were blinded to group allocation. The flow of participants through the recruitment process and randomisation is presented in Figure 1.

### The intervention

Participants randomised to Group 1 received a 30 minute physical activity consultation with a trained member of the research team. The transtheoretical model of behaviour change was used as a theoretical framework for the consultation and followed recommended guidelines [14]. This consultation focused on uptake of physical activity, discussion of barriers and formation of goals incorporating the walking programme. This approach has previously been used to show successful physical activity behaviour change [19-21]. The participant was given an individualised 12 week walking programme and a pedometer. The aim of the walking programme was for participants to increase their average daily step count by 3,000 steps above their baseline value on at least five days of the week by week 6 and maintain this to week 12. The 3,000 steps value is based on the assumption that an adult walking at a moderate pace takes 100 steps/minute (1,000 steps/10 minutes) [22]. An increase of 3,000 steps/day would correspond to an increase of approximately 30 minutes of moderate physical activity, i.e. the physical activity recommendation for adults.

Following the 12 week walking programme, the participants received a second individual physical activity consultation framed according to the transtheoretical model. This consultation focused on relapse prevention, encouragement and maintenance of activity. Participants received a written physical activity advice leaflet at 24 weeks and a telephone consultation at 36 weeks.

Participants randomised to Group 2 were allocated to a 12 week waiting list and were requested not to amend their current physical activity levels. After 12 weeks Group 2 received an individualised 12 week walking programme identical to Group 1, brief advice and a pedometer but did not receive a physical activity consultation (i.e. the waiting list control group then became a minimal intervention group). At 24 weeks (end of their programme) and 36 weeks (equivalent to the time when group 1 received the advice leaflet) participants received a short feedback session. Nothing further was given to this group until they were recalled after 60 weeks.

### Setting

Interviews, physical activity consultations and completion of questionnaires took place in a specially allocated study room within a University building. Physiological assessments took place in a University laboratory.

### Data management

Study data were entered in a customised Microsoft Excel database and stored on a secure network drive. All behavioural, psychological, physiological and subjective environmental data were double entered and cross checked by a different member of the research team. Paper records were stored in a secure location.

#### Individual Studies

##### i) Behavioural Study

Walking behaviour was assessed using two methods. The primary outcome measure was pedometer step counts (Omron HJ-109E Step-O-Meter). The secondary outcome measure was 7-day recall of physical activity using the International Physical Activity Questionnaire (IPAQ), (long version, self-report) [23]. The behavioural impact of the intervention was assessed over a 12 month period. This enabled evaluation of the short term, immediate effect of the intervention and also whether the intervention resulted in a longer term, sustainable change in behaviour. In Group 1 walking behaviour was assessed at baseline, 12 weeks, 24 weeks and 48 weeks. In Group 2 walking behaviour was assessed at baseline, 12 weeks, 24 weeks, 36 weeks and 60 weeks.

##### ii) Transtheoretical Model

The Transtheoretical Model was used as a theoretical framework to investigate the relationship between participants' **psychological **constructs and behaviour change. Specifically, the study examined whether any of the four constructs of the Transtheoretical Model (stages of change, processes of change, self efficacy, and decisional balance [24]), along with mood (Positive and Negative Affect Schedule (PANAS) [25]) and quality of life (Euroqol EQ-5D [26]) predicted behaviour change, and if behaviour change had a consequential effect on these variables. In Group 1 these questionnaires were completed at baseline, 12 weeks, 24 weeks and 48 weeks. In Group 2 the questionnaires were completed at baseline, 12 weeks, 24 weeks, 36 weeks and 60 weeks.

##### iii) Physiological Study

The WWW study investigated the **physiological **response to the intervention in terms of body composition, blood pressure, heart rate, total cholesterol, high density lipoprotein (HDL)-cholesterol, insulin and glucose, and also investigated the impact of increased walking on circulating levels of inflammatory markers. In recent years clear evidence has emerged of the involvement of inflammatory mechanisms in several diseases including cardiovascular disease [27], colorectal cancer [28], stroke [29], obesity [30] and type 2 diabetes [30]. With 65% of men and 60% of women in Scotland categorised as overweight [6], 3% diagnosed with type 2 diabetes [31], and death rates from coronary heart disease the second highest in Western Europe [32], a greater understanding of possible interventions is a key public health goal. Chronic low grade inflammation can be defined as 2–4 fold elevations in both pro- and anti-inflammatory cytokines at rest [33] and regular exercise has been shown to decrease resting levels of key inflammatory markers [34]. WWW therefore investigated whether regular walking can decrease resting levels of three key inflammatory cytokines (interleukin-6 (IL6), C-reactive protein (CRP) and tumour necrosis factor α (TNF-α)) and their receptors (sIL-6R, TNFα, TNFαR1 and TNFαR2).

In Group 1 all physiological measures were taken at baseline and 12 weeks. At 24 weeks body mass, BMI, waist-to-hip ratio, percentage body fat, blood pressure and heart rate were assessed. In Group 2 all physiological measures were assessed at baseline, 12 week and 24 weeks. At 36 weeks body mass, BMI, waist-to-hip ratio, percentage body fat, blood pressure and heart rate were assessed. Full details of how these measures were obtained are in a separate paper (Baker *et al*., submitted for publication).

##### iv) Environmental Study

The physical **environment **can facilitate or inhibit physical activity across populations. Within neighbourhoods, factors such as aesthetics, convenience of facilities, accessibility of destinations and perceptions of traffic safety have been shown to be associated with levels of walking [35]. Psychosocial variables may also influence this relationship [36]. The WWW study investigated the relationships between physical activity levels, in particular walking, and perceived (subjective) environmental barriers or facilitators to activity, and also any changes in physical activity levels and environmental perceptions over the course of the study. Self-reported perceptions of the physical environment can change over a relatively short period of time and this may be associated with a change in the level of moderate-intensity physical activity [37]. The change may not always occur in a positive direction but evidence suggests that those who are already active report the most positive perceptions of the environment [38]. The Neighbourhood Quality of Life Study (1^st ^Survey) (NQLS) was used to subjectively assess the participants' perceptions of their local environment in relation to physical activity. The NQLS incorporates 7 subscales of the Neighbourhood Walking Scale (NEWS) and 5 subscales that assess psychosocial variables related to the neighbourhood environment and physical activity behaviour. The NQLS psychosocial subscales are:

1. Enjoyment of physical activity (developed by the NQLS group)

2. Benefits of exercise (adapted from Hovell *et al *[39] and Calfas *et al *[40])

3. Social support for physical activity: Acceptable test-retest and internal consistency reliabilities and evidence of concurrent criterion-related validity [41]

4. Barriers to regular physical activity (adapted from Hovell *et al *[39] and Calfas *et al *[40])

5. Social cohesion of neighbourhood: The social cohesion subscale is a 5 item measure of collective efficacy that has been shown to yield high between-neighbourhood reliability [42].

The NEWS survey items have been adapted for use in a Scottish population (for example, replacing the word condominiums with the word tenements and removing references to canyons in the neighbourhood). This adapted form of the NEWS has previously been used with Glaswegian adolescents (Hamilton, L., unpublished undergraduate thesis). Two additional sections were added to the questionnaire to consider the effects of other barriers (i.e. weather) and also to investigate respondents' perception of distance.

In Group 1 these questionnaires were completed at baseline, 12 weeks, 24 weeks and 48 weeks. In Group 2 the questionnaires were completed at baseline, 12 weeks, 24 weeks, 36 weeks and 60 weeks.

An environmental audit tool has been developed and used to objectively assess the WWW study area, based on the SPACES audit tool developed by Pikora *et al *[43]. The survey items were adapted in this WWW project for use in a Scottish urban context. Surveying the study area using the audit tool enabled the walkability of an area around each participant's home that can be accessed within approximately 30 minutes' total walking time (radius of 1.6 km, as used by Giles-Corti *et al *[44]) to be assessed, as well as assessment of particular local walking routes described by the participants. The audit tool included aspects of the physical environment that have been demonstrated to be correlated with physical activity and particularly walking, for example path quality [45], access to destinations such as shops, recreational facilities, parks and public transport stops [45-47], aesthetics [46-48] and safety [48,49], as well as additional aspects that seem likely to be influential in the UK context, e.g. pavement width. Residential density, land use mix and street connectivity have also been correlated with physical activity [50] and these have been calculated using GIS to complement the findings of the environmental audit.

##### v) Qualitative Study

To understand the social context of the WWW study, **qualitative **research was undertaken alongside the randomised trial. This provided an insight into awareness of the project in the local community (through semi-structured interviews with general practitioners, shop-keepers and library staff), an insight into levels of interest among the target population (through observation carried out at key locations) and an insight into participants' experiences of and attitudes towards the walking intervention (through a series of focus groups). In the focus group discussions, an attempt was made to identify both the barriers and aids to adherence to the walking programme and to highlight any differing experiences for men and women. In addition, semi-structured interviews with members of the research team captured their experiences of the study and their thoughts on the feasibility of implementing the intervention.

### Supplementary study

In addition to the studies that were planned from the outset, an additional study supplemented the WWW project. This is detailed below.

#### Economic evaluation of the intervention

An economic evaluation was undertaken using the participant level data from the trial. The costs included were the short term costs of the intervention (pedometer, consultation etc.) plus any differences in costs resulting from changes in NHS resource use between the intervention and control group. Unit costs based on study specific estimates, or derived from published sources (Unit Costs of Health and Social Care and Scottish Health Service Costs), were combined with estimates of resource use to determine total costs. EQ-5D, administered to Group 1 at baseline, 12 weeks, 24 weeks and 48 weeks and baseline, 12 weeks, 24 weeks, 36 weeks and 60 weeks in Group 2 was used to determine the quality of life for the intervention and control groups. This was converted into a within trial estimate of quality adjusted life years (QALY) using the area under the curve method. In the primary analysis, costs were compared to QALY, measured within trial, to give cost-effectiveness in terms of cost/QALY gained. A subsequent analysis examined the cost per individual achieving the assumed target (30 minutes of physical activity on 5 days/week).

### Type of analysis used including a power calculation

Data analysis is on-going. A multi-method approach is being adopted. Quantitative outcome measures are being analysed using appropriate univariate and multivariate techniques. Analysis of quantitative data is on an intention to treat basis (with the exception of some of the physiological markers). Qualitative data is being thematically analysed. Thematic analysis is a method for "identifying, analysing and reporting patterns (themes) within data"[51]. Essentially it involves coding participants' talk into categories that summarise and systemise the content of the data.

G-Power analysis [52] set for F-test ANOVA was used to calculate sample size for between group analyses of the primary outcome measure (daily step count). Power was set at 0.8, Alpha level was set at 0.05 and effect size (Cohen's *f*) was set at 0.4 (large) [53] for the two group (intervention and control) design. A minimum sample size of 52 was calculated (26 participants in each group respectively).

Statistical power was also calculated for the major inflammatory marker, IL-6. With two groups (intervention and control) repeated measures study design, a correlation between trials of 0.85, a significance level of 0.05 and an n of 23 in each group, this study would have a power of 0.80 of detecting a medium interaction effect (0.5) [54]. A standardised medium effect size of 0.5 equates to an absolute decrease in IL-6 levels of 0.30 pg/ml. This effect size was chosen on the basis of findings published by You *et al. *[55] who found an absolute decrease in IL-6 levels of 0.48 pg/ml in response to a 24 week intervention of diet plus exercise. Therefore in this study of a 12 week exercise intervention a decrease in IL-6 levels of 0.30 pg/ml seems a reasonable estimate. A similar analysis was also calculated based on the total cholesterol/HDL ratio. With two groups (intervention and control) repeated measures study design, a correlation between trials of 0.85, a significance level of 0.05 and an n of 25 in each group, this study would have a power of 0.80 of detecting an interaction effect (0.35). This effect was calculated from the absolute decrease of 0.3 found by Kelly *et al.*, 2004 [56].

## Discussion

Due to report in 2008, WWW addresses several of the evidence gaps in the physical activity literature in relation to walking that were identified by Ogilvie *et al*., (2007) [8]. WWW, a multi-disciplinary RCT, was designed to assess the effectiveness of a community based walking programme using pedometers in combination with physical activity consultations at increasing and sustaining walking behaviour over 12 months. Study participants were drawn from a 'real world' sample from a local community. The decision to aim for this group was informed by the RE-AIM principles [57]. The study has six key research components: (behavioural, psychological, environmental, physiological, qualitative and economic) allowing an insight into the complex relationships between behavioural change, health consequences and the role of the environment, along with participants' views and experiences and the cost effectiveness of this approach.

An on-going issue with physical activity research is appropriate terminology to classify activity levels (e.g. sedentary, low active, active) and clear definitions for these terms in relation to both objective measures and subjective measures. Cultural differences in activity levels may result in regional variations in terminology. A strength of WWW is the assessment of physical activity using both objective (pedometer step counts) and subjective (IPAQ physical activity recall questionnaire) measures. The study may suffer from three limitations of internal validity common to physical activity interventions: blinding participants to their allocation status, misclassification of physical activity and using personnel to collect main outcome measures that were independent and blinded to group allocation [58]. However blinding to allocation status is very difficult in a physical activity intervention and more appropriate to a pharmacological study. The insensitivity of self reported physical activity measures leads to less precision in its measurement and increases the variance in measures of behaviour. As intervention and control group participants completed the same self report measure, any misclassification is likely to be non-differential leading to an attenuation of the effect of the intervention. We also attempted to blind outcome measures from study personnel where appropriate. A final limitation of the trial is the lack of a control group for the whole duration of the study (waiting list control group were given a minimal intervention after 12 weeks). Due to the well established relationship between physical activity and health we felt it was unethical not to provide all participants with the opportunity to increase their walking behaviour.

The environmental research element of WWW includes both subjective and objective measures. The use of subjective and objective environmental measures combined is strongly recommended to maximise capture of the greatest number of physical activity domains and to improve the predictive capacity of future studies [59]. It also allows perceptions of environmental barriers and facilitators to walking to be set against objective measures and precise descriptors of the physical attributes of the environment that can form the basis of guidance to planners and designers of the environment. To date environmental audit tools have been developed primarily for use in American or Australian environments. These instruments have obvious limitations for use when applied to other countries. The WWW audit tool has been developed specifically for use in the study area, enabling an objective environmental assessment in relation to physical activity to be carried out in the UK.

As WWW is a multi-disciplinary trial, the development and implementation required a large team of researchers. Regular team meetings, a trial co-ordinator and hands-on leadership helped to address the management issues associated with such a trial. The WWW trial is one element of the work of SPARColl (Scottish Physical Activity Research Collaboration, ). The SPARColl Advisory group comprises seven physical activity experts each of whom contributed their expertise to the conceptualisation and design of WWW (NM, MN, CWT, JI, DO, CEF and Fiona Bull).

## Competing interests

The authors declare that they have no competing interests.

## Authors' contributions

All authors were involved in conceptualisation and design of the study and commented on the writing of the manuscript. CFF drafted the manuscript and was the trial co-ordinator. NM was the principal investigator and led the study. NM, GB, AW and RL designed and quality assured the walking intervention and devised the assessment of physical activity. GB and AW performed the physical activity consultations. NM, AW and RL designed the subjective environmental assessments. CWT and CM designed the objective environmental assessment. NM and GB designed the psychological assessments. MAN and CFF designed the physiological assessments. RS designed the qualitative components. EF designed the economic evaluation.

## Pre-publication history

The pre-publication history for this paper can be accessed here:


